# Folic acid supplements and perinatal mortality in China

**DOI:** 10.3389/fnut.2023.1281971

**Published:** 2024-01-08

**Authors:** Xiaojing Liu, Xiaowen Liu, Hang An, Zhiwen Li, Le Zhang, Yali Zhang, Jianmeng Liu, Rongwei Ye, Nan Li

**Affiliations:** ^1^Department of Epidemiology and Biostatistics, School of Public Health, Peking University Health Science Center, Beijing, China; ^2^Institute of Reproductive and Child Health/Ministry of Health Key Laboratory of Reproductive Health, Peking University Health Science Center, Beijing, China

**Keywords:** folic acid supplementation, perinatal mortality, stillbirth, early neonatal death, neonatal death

## Abstract

**Introduction:**

Periconceptional use of multivitamins containing folic acid prevents external major birth defects, especially neural tube defects. We aimed to explore the effects of maternal folic acid supplementation alone on perinatal mortality with or without external major birth defects plus neural tube defects.

**Methods:**

From the China-US Collaborative Project for Neural Tube Defects Prevention, we identified 222, 303 singleton pregnancies with detailed information on periconceptional folic acid use, defined as folic acid supplementary before the last menstrual date until to the end of the first trimester. Perinatal mortality included stillbirths after 20 weeks’ gestation and early neonatal deaths within 7 days of delivery.

**Results:**

Among the fetuses or infants of women who did not take folic acid, the rate of perinatal mortality was 2.99% and 1.62% at least 20 weeks’ gestation in the northern and southern regions. Among the fetuses or infants of the women with periconceptional use of folic acid, the rates were 1.85% and 1.39% in the northern and southern region. The estimated relative risk for perinatal mortality [adjusted risk ratio (RR), 0.72; 95% confidence interval (CI), 0.61- 0.85], stillbirth (adjusted RR, 0.78; 95% CI, 0.64-0.96), early neonatal mortality (adjusted RR, 0.61; 95% CI, 0.45-0.82), and neonatal death (adjusted RR, 0.64; 95% CI, 0.49-0.83) in northern China was significantly decreased in association with periconceptional folic acid supplementation. Compared with northern, there was a lesser effect in southern China.

**Conclusion:**

Periconceptional intake of 400μg folic acid daily reduces the overall risk perinatal mortality, as well as the risk from external major birth defects and neural tube defects, especially in northern China.

## 1 Introduction

Perinatal mortality is one of the most noticeable adverse pregnancy outcomes, and causes millions of deaths each year, especially in developing countries (1–3). Perinatal mortality is categorized into stillbirth and early neonatal death according to clinical manifestation. A total of 2.65 million babies is stillborn annually, with 98% occurring in low- and middle-income counties. The etiology of perinatal mortality is multifactorial but remains unclear.

Women’s nutritional intake during pregnancy may play a vital role in maternal morbidity and mortality (4, 5), although few data exist on the effect of nutritional supplementation on perinatal mortality. A 2011 meta-analysis and a 2013 systematic review do not found protective effect of folic acid-fortified multivitamin on perinatal mortality (6, 7). However, they do not take folic acid supplements alone into consideration, and its potential role in preventing perinatal mortality remains unclear. Existing evidence supported that structural births defects, especially external major birth defects and neural tube defects, were common and contributed to perinatal mortality (8, 9). Further a systemic meta-analysis study showed folic acid supplementation could prevent against neonatal mortality by decreasing the incidence of neural tube defects (10); the most recent review of the correlation between folic acid and all-cause perinatal mortality was based on observational studies that cannot distinguish whether effects on perinatal mortality are mediated by external major birth defects and neural tube defects or are simply concurrent. However, the public health impact of the reduced perinatal mortality should be further evaluated by assessment of external major birth defects and neural tube defects.

The only antenatal supplement promoted by the Chinese Ministry of Health and United States Public Health Service is 400 μg of folic acid daily with no additional vitamins to prevent neural tube defects during the 1990s (11), which has been adopted by other countries (12). Several cohort studies have confirmed the effectiveness of this campaign (13–15). With the large and prospective birth cohort in China, we aimed to explore the association of periconceptional supplementation with folic acid alone and the risk of perinatal mortality overall or certain compositions thereof, and further to examine this association with or without major external birth defects and neural tube defects in this study.

## 2 Methods

### 2.1 Background of the cohort

The methods of the original study have been described previously (13, 16). Beginning in 1993, the Chinese Ministry of Health conducted a public health campaign to prevent neural tube defects in 21 counties in two southern provinces (Zhejiang and Jiangsu) and one northern province (Hebei). During this campaign, all couples planning to marry undergo a premarital examination in China. All women in the three provinces who were pregnant and who prepared to get marry were registered in a pregnancy monitoring system, which was linked to the detailed data about demographic information, folic acid supplement, and perinatal health care record. The perinatal health care record was designed to monitor the course and outcome of all the resident women. All women were advised to take a pill containing 400 μg of folic acid alone every day from the registration time to the end of the first trimester of pregnancy. If women consented to take folic acid, the pills were distributed at the time of registration. At the end of each month, the health workers recorded the dates of all menstrual periods and how many pills remained in each bottle. The original cohort comprised 247,831 women who registered with the pregnancy-monitoring system between October 1993 and September 1995 and who delivered by December 31, 1996. The project was approved by the institutional review boards of the Centers for Disease Control and Prevention and Beijing Medical University. All women who took pills provided verbal informed consent.

### 2.2 Definition of folic acid use

Women who took folic acid pills at any time from the registration period until the end of the first trimester of pregnancy were classified as folic acid users. Folic acid usage was divided into three patterns based on the usage period: (i) periconceptional use, defined as the initiation of folic acid supplementation before the last menstrual period and termination at the end of the first trimester; (ii) preconceptional use, defined as the initiation and termination of folic acid use before the last menstrual period; and (iii) postconceptional use, defined as the initiation of folic acid supplementation after the last menstrual period but within the first trimester. Women who did not agree to take folic acid or who were registered during the second trimester of pregnancy were considered to be non-users. Compliance was calculated for each woman by dividing the total number of pills taken by the total number of days between the initiation and termination of supplementation.

### 2.3 Case ascertainment of stillbirth, early neonatal deaths, neonatal deaths, and major external birth defects

The detailed data about fetuses or infants with stillbirth, early neonatal deaths, neonatal deaths, and external structural birth defects were obtained by a birth-defects surveillance system that was established in January 1993. The atlas contains detailed descriptions, photographs, and International Classification of Diseases, Ninth Revision, codes for 26 common birth defects (17). Birth defects that are not included in the atlas are coded as ‘unknown’. The number and frequency of each type of major external birth defects excluded in the present study were provided in Supplementary Table 1. Three pediatricians independently reviewed the report and photographs and assigned diagnostic codes. Neonatal deaths were circumscribed as live births registered as having died within the first 28 days of life. We defined perinatal mortality as stillbirths (fetuses delivered at 20 weeks’ gestation or later with no signs of life and recorded as occurring before the onset of or during labor) plus early neonatal deaths (deaths among liveborn infants occurring within 7 days of delivery) (18).

### 2.4 Statistical analysis

We compared the characteristics of maternal age, body mass index (BMI), ethnicity, education, occupation, and parity between women who took folic acid and those who did not. We compared the means of age and BMI using *t*-tests, and the distributions of categorical variables using chi-square tests. Using the logistic regression model, we estimated risk ratios (RRs) by dividing the incidence of perinatal mortality among women who took folic acid by that among women who did not; we also estimated risk reduction to observe the different effect of folic acid on perinatal mortality between the northern and southern China. Both unadjusted and adjusted RRs were derived after adjustment for potential confounders including maternal age at delivery (continuous), BMI (continuous), ethnicity, parity, education, and occupation. The participants were divided into three subgroups to compare the differences in effects of the timing and compliance of folic acid use on perinatal mortality. We used mean imputation to substitute missing values of those confounding variables in the logistic regression. Findings at *p* < 0.05 were considered significant. All analyses employed SPSS ver. 20.0 software (SPSS, Inc., Chicago, IL, USA).

## 3 Results

After the exclusion of pregnant women who were lost to follow-up or those for whom the status of the fetus or infant with respect to perinatal mortality was unknown, there were 222,303 singleton pregnant women (28,829 in the northern region and 193,474 in the southern region). The proportion of folic acid supplementation was higher in the north (58.9%) than the south (52.9%). The difference analysis results of demographic characteristics showed the women who took folic acid pills were approximately 2 years younger than those who did not, and were more likely to be primiparous, factory workers, and better educated in both regions (Table 1).

**Table 1:** Characteristics of women who enrolled in the pregnancy monitoring system according to folic acid use, China, 1993 to 1996.

	North					South				
Characteristic	Folic acid users (*n* = 16,974)		Nonusers (*n* = 11,855)		*P*	Folic acid user (*n* = 102,333)		Nonusers (*n* = 91,141)		*P*
	*n*	%	*n*	%		*n*	%	*n*	%	
Age (years, mean [SD])	24.52 (2.49)		26.90 (4.02)		<0.001	24.75 (2.42)		26.05 (3.74)		<0.001
Body mass index (kg/m^2^, mean [SD])	21.15 (2.01)		21.12 (1.52)		0.118	20.33 (2.13)		20.67 (1.86)		<0.001
Primiparous	16,222	95.57	7,359	62.08	<0.001	93,853	91.71	65,400	71.76	< 0.001
Han ethnic group	16,525	97.35	11,665	98.40	<0.001	101,366	99.06	90,330	99.11	0.205
Education					<0.001					<0.001
High school or higher	1,513	8.91	887	7.48		11,450	11.19	8,674	9.52	
Junior high school	12,920	76.12	9,255	78.07		64,105	62.64	49,977	54.83	
Primary school or lower, or unknown	2,541	14.97	1713	14.45		26,778	26.17	32,490	35.65	
Occupation					<0.001					<0.001
Farmer	14,669	86.42	10,853	91.55		54,416	53.18	60,789	66.70	
Factory worker	1,619	9.54	743	6.27		42,230	41.27	25,927	28.45	
Other or known	686	4.04	259	2.18		5,687	5.56	4,425	4.86	

SD, standard deviation.

According to the results of logistic regression model, there were significant differences in the associations between folic acid supplementation with perinatal mortality occurrence in the north and south of China (Table 2). After adjusting for maternal age, BMI, education, occupation, ethnicity, and parity, folic acid supplementation significantly prevented perinatal mortality (adjusted RR: 0.72, 95% CI: 0.61–0.85) in northern China. A lesser preventive effect of folic acid on perinatal mortality (adjusted RR: 0.92, 95% CI: 0.85–0.99) was observed in southern China. Additional adjustment for gestational age and birth weight did not change the slight disproportion in the north and south of China (data not shown). When we further classified the cases of perinatal mortality into subgroups according to whether birth defect was unaccompanied by additional major external anomalies or neural tube defects, all differences were significant in northern and southern China.

**Table 2:** Association of folic acid use with perinatal mortality in China, 1993 to 1996 per 1,000 births.

Folic acid use	North			South			Risk reduction^c^		
	All cases of perinatal mortality	All cases without major external birth defects	All cases without neural tube defects	All cases of perinatal mortality	All cases without major external birth defects	All cases without neural tube defects	All cases of perinatal mortality	All cases without major external birth defects	All cases without neural tube defects
	No. (Rate, %)	No. (Rate, %)	No. (Rate, %)	No. (Rate, %)	No. (Rate, %)	No. (Rate, %)	RD (95% CI)	RD (95% CI)	RD (95% CI)
None	11,855 (29.9)	11,668 (21.1)	11,779 (24.5)	91,141 (16.2)	90,405 (12.9)	91,063 (15.5)			
Use	16,974 (18.5)	16,871 (15.9)	16,952 (17.5)	102,333 (13.9)	101,594 (11.0)	102,271 (13.3)			
RR 95% CI	0.61 0.53, 0.71	0.75 0.63, 0.89	0.71 0.60, 0.84	0.86 0.80, 0.92	0.85 0.78, 0.92	0.86 0.80, 0.93	0.33 (0.18, 0.49)	0.13 (−0.05, 0.30)	0.19 (−0.02, 0.36)
Adjusted RR 95% CI	0.72 0.61, 0.85	0.80 0.66, 0.97	0.79 0.66, 0.94	0.92 0.85, 0.99	0.90 0.83, 0.98	0.92 0.85, 0.99	0.24 (0.05, 0.43)	0.11 (−0.11, 0.34)	0.16 (−0.05, 0.36)
P^b^	<0.001	0.026	0.008	0.027	0.019	0.037	0.015	0.323	0.141

No., number of pregnancies; RR, risk ratio; CI, confidence interval; RD, risk differences.

^a^Adjusted for maternal age (continuous), BMI (continuous), education, occupation, folic acid use, ethnicity, and parity.

^b^Adjusted RR.

^c^Risk reduction of perinatal mortality in the north when compared with that in the south.

We compared the differences in the associations between folic acid use and perinatal mortality by the timing and compliance of taking folic acid (Table 3). The prevention effect of periconceptional folic acid use in northern China (adjusted RR: 0.62, 95% CI: 0.51–0.75) was more obviously than that in southern China (adjusted RR: 0.90, 95% CI: 0.82–0.98). However, folic acid use before or during pregnancy was not associated with a reduced risk of perinatal mortality in either northern or southern China. The protective effect increased in magnitude with higher folic acid usage compliance. The risk reduction of ≥90% folic acid usage compliance tended to be 20% higher for women from northern China than for those from southern China.

**Table 3:** Timing of, and compliance with, folic acid supplementation and risk of perinatal mortality in China, 1993 to 1996 per 1,000 births.

Folic acid use	All cases of perinatal mortality in the north				All cases of perinatal mortality in the south				RD^c^ (95% CI)
	No.	Rate^a^	Crude RR (95% CI)	Adjusted RR (95% CI)^a^	No.	Rate^a^	Crude RR (95% CI)	Adjusted RR (95% CI)^a^	
Timing^b^									
Periconception	11,983	15.9	0.53 (0.44, 0.63)	0.62 (0.51, 0.75)	54,410	13.5	0.83 (0.76, 0.91)	0.90 (0.82, 0.98)	0.37 (0.16, 0.58)
Preconception	3,395	25.6	0.85 (0.67, 1.08)	1.00 (0.78, 1.28)	32,725	14.0	0.87 (0.78, 0.96)	0.92 (0.83, 1.03)	−0.09 (−0.35, 0.18)
Postconception	1,568	23.0	0.76 (0.54, 1.08)	0.88 (0.62, 1.25)	15,132	14.4	0.89 (0.77, 1.03)	0.95 (0.82, 1.10)	0.05 (−0.34, 0.43)
Compliance									
<70%	1,663	19.2	0.64 (0.44, 0.92)	0.73 (0.50, 1.06)	4,352	16.3	1.01 (0.80, 1.28)	1.08 (0.85, 1.37)	0.38 (−0.02, 0.78)
70% to <90%	3,839	19.0	0.63 (0.49, 0.81)	0.73 (0.56, 0.95)	12,945	14.2	0.88 (0.75, 1.03)	0.94 (0.81, 1.10)	0.24 (−0.07, 0.56)
≥90%	11,472	18.2	0.60 (0.51, 0.72)	0.71 (0.59, 0.86)	85,036	13.7	0.85 (0.78, 0.91)	0.91 (0.84, 0.98)	0.24 (0.02, 0.46)

No, number of pregnancies; RR, risk ratio; CI, confidence interval; RD, risk differences.

^a^Adjusted for maternal age (continuous), BMI (continuous), education, occupation, ethnicity, and parity.

^b^The analysis excluded 28 and 66 women in the northern and southern regions, respectively, for whom the timing of folic acid use could not be classified.

^c^Risk reduction of perinatal mortality in the north when compared with perinatal mortality risk in the south after adjusting the potential confounders.

Table 4 showed the effects of folic acid on the three main compositions of perinatal and neonatal death in northern and southern China. The lesser risk associated with folic acid pill use was more obvious in the north than the south. In the north, there was a 20 to 30% reduced risk of stillbirth, early neonatal death, and neonatal death among women taking folic acid pills. In the south, there were no significant associations between folic acid use and reduced risk of stillbirth (adjusted RR: 0.96, 95% CI: 0.87–1.07) and neonatal death (adjusted RR: 0.92, 95% CI: 0.83–1.02), except for early neonatal death (adjusted RR: 0.86, 95% CI: 0.77–0.97).

**Table 4:** Association of folic acid use with perinatal mortality composition in China, 1993 to 1996 per 1,000 births.

	North			South			Risk reduction^c^		
Folic acid use	Stillbirth	Early neonatal death	Neonatal death	Stillbirth	Early neonatal death	Neonatal death	Stillbirth	Early neonatal death	Neonatal death
	No. (Rate, %)	No. (Rate, %)	No. (Rate, %)	No. (Rate, %)	No. (Rate, %)	No. (Rate, %)	RD (95% CI)	RD (95% CI)	RD (95% CI)
None	11,855 (20.2)	11,616 (9.9)	11,616 (12.6)	91,141 (9.2)	90,305 (7.0)	90,305 (8.9)			
Use	16,974 (13.1)	16,752 (5.5)	16,752 (7.0)	102,333 (8.2)	101,498 (5.7)	101,498 (7.7)			
RR 95% CI	0.64 0.54, 0.77	0.55 0.42, 0.73	0.55 0.43, 0.71	0.89 0.81, 0.98	0.82 0.73, 0.91	0.87 0.79, 0.96	0.32 (0.11, 0.53)	0.39 (0.10, 0.68)	0.45 (0.18, 0.72)
Adjusted^a^ RR 95% CI	0.78 0.64, 0.96	0.61 0.45, 0.82	0.64 0.49, 0.83	0.96 0.87, 1.07	0.86 0.77, 0.97	0.92 0.83, 1.02	0.20 (0.02, 0.39)	0.34 (0.01, 0.67)	0.36 (0.04, 0.68)
*P* ^b^	0.018	0.001	0.001	0.464	0.012	0.097	0.034	0.041	0.029

No., number of pregnancies; RR, risk ratio; CI, confidence interval; RD, risk differences.

^a^Adjusted for maternal age (continuous), BMI (continuous), education, occupation, ethnicity, and parity.

^b^Adjusted RR.

^c^Risk reduction of perinatal mortality composition in the north when compared with perinatal mortality risk in the south.

We compared the differences in preventive effect of folic acid supplementary on perinatal mortality between different regions. When compared with the south, there were significant risk reductions in all cases of perinatal mortality (Table 2), periconception use of folic acid, usage compliance ≥90% (Table 3), and perinatal mortality composition (Table 4) in the north. We additionally excluded the cases with major birth defects (Supplementary Tables S2, S3) or neural tube defects (Supplementary Tables S4, S5) from perinatal mortality, to analyze the effects of overall folic acid use (Supplementary Tables S3, S5), usage time and compliance (Supplementary Tables S2, S4). The results showed the preventive effects on perinatal mortality were larger in the northern region of China, periconceptional folic acid use, and higher compliance compared with the corresponding reference.

## 4 Discussion

In this large population-based cohort study, we observed a substantially decreased risk for perinatal mortality with or without external major birth defects plus neural tube defects, especially neonatal deaths, among pregnant women who took folic acid supplements during the periconceptional period compared with those who did not. The protective association for perinatal mortality and neonatal deaths tended to be greater in the north than the south, and greatest among women with greater compliance with folic acid supplementation. Our study provides further support for the hypothesis that folic acid supplementation prevents perinatal mortality in populations with low folate concentrations.

There was a marked difference in the risk of perinatal mortality between the north and the south. The protective effect was greater among women in northern China than southern China. One possible explanation for this geographic difference is that diets in the north were more likely to be folate deficient. Based on the overall higher socioeconomic status and generally greater availability of fresh vegetables in the southern region, diets in the south were likely richer in folate than those in the north. As a whole, the southern region (which is adjacent to Shanghai) is one of the wealthiest regions in China and has a more temperate climate with a longer growing season than the northern region. In 2003, our colleagues randomly selected one county and one city in the north and south from the same regions as this study and examined the first-trimester folate concentration in blood samples. Women in the north (440.0 nmol/L) had less than half the red blood cell folate levels of women in the south (910.4 nmol/L) (19). This is supported by data from a cross-sectional study showing a significantly higher frequency of folate deficiency in the north (40%) compared with the south (6%), based on plasma and red-blood-cell folate concentrations (20).

We estimate that folic acid supplementation has the potential to prevent about 20% of perinatal mortality, partly due to visible congenital malformations. A large prospective cohort study in China showed that periconceptional supplementation with folic acid alone reduces the occurrence of neural tube defects (13), and similar findings have been reported around the world (21, 22). As shown by a meta-analysis based on eight population-based observational studies, folic acid food fortification could give an estimated reduction in neural tube defect incidence of 46%, and then prevent 13% of neonatal deaths associated with visible congenital defects (9). Moreover, we found folic acid was significantly correlated with decreased risk of 26 common visible congenital birth defect and central nervous system defect (Supplementary Table S6). The preventive effect on external major birth defect in the present study were consistent with previous findings (14, 23, 24). We further found the preventive effect for central nervous system defect was stronger than neural tube defect. That means folic acid supplementary also has potential role in other varieties of nervous system defect except for neural tube defect, but the extents to which need more intervention studies to verify.

A healthy nutrition status during pregnancy was correlated with infant morbidity and mortality, partly resulting from congenital malformations (25, 26). But women who supplemented with multiple micronutrients containing folic acid or iron-folic acid could not significantly reduce the risk of perinatal mortality based on our team’s *post hoc* analysis study (27) and other two trials in Nepal (28, 29). We previously have found folic acid supplements have a potential possibility to promote fetal growth with increasing birth weight and gestation duration (25). However, we did not find that gestational age and birth weight could affect the preventive effect of folic acid supplement on perinatal mortality. There has been well-established evidence on the benefit of folic acid for external birth defect reduction (30, 31). As for internal birth defect, it’s also confirmed that folic acid supplementary can effectively prevent against congenital heart defect, respiratory system and digestive system defect, and then reduce the occurrence of perinatal mortality (32–34). It can be speculated the reduced risk of perinatal mortality associated with folic acid may be partly mediated by low incidence of internal birth defect. More additional evidence is needed to clarify possibly indirect role of fetal growth and nutrition status among women who supplement with folic acid in prevention of perinatal mortality. The association of specific compositions of perinatal mortality have been inconsistent, potentially because of the small numbers of cases and different study designs, populations, and methods of classifying cases (27, 35, 36). We found a significant preventive effect of folic acid on peri- and early neonatal mortality in both northern and southern China. Different preventive effects of folic acid on different compositions of perinatal mortality with or without external major birth defects were observed. These observations suggest that the folate-mediated, single-carbon metabolic pathways through which folic acid acts to reduce perinatal mortality risk may be nonspecific and that folic acid protects against perinatal mortality in part by suppressing external major birth defects. Our finding that maternal folic acid supplementation reduces the risk of perinatal mortality with or without other anomalies/neural tube defects is consistent with this notion. In this evaluation, women were not randomly selected to take or not to take folic acid pills. Therefore, the women who took them may have differed systematically from those who did not in factors that influence the birth order. Compared with the women who took folic acid, those who did not were more likely to have been pregnant before. Our results emerged into unobvious association between folic acid supplementation and stillbirth or neonatal death after taking into account potential confounders. Stratification according to the number of previous pregnancies, however, did not change the results.

The strengths of our evaluation were its use of a population-based prospective cohort design, with nearly complete ascertainment of outcomes, and that we prospectively documented the monthly recording of folic acid use during the period of gestation before the pregnancy outcome was known. Second, the only intervention was folic acid supplementation, so the results reflect the effect of folic acid alone. The dose of folic acid was 0.4 mg, and we identified effects associated with this dose. At the time, few women in the study area could afford multiple vitamins, and there was little market supply of multiple vitamins. The study period was before implementation of the national folic acid supplementation program, and so the results may reflect the effect of folic acid concentrations. Furthermore, we adopted an appropriate prospective surveillance system for birth defects, which was established before the evaluation commenced and identified all affected fetuses and infants. The well-organized monitoring system included all births at 20 complete gestational weeks (including live births, stillbirths, and pregnancy terminations) and all structural congenital anomalies irrespective of gestational week (13). This will facilitate more precise estimations of birth defects than those provided by hospital-based surveillance systems, because some external structural birth defects are terminated following prenatal diagnosis before 28 gestational weeks in China (37). This study used quality controls to ensure data quality by establishment of diagnoses on the basis of photographs taken at birth and reviews of detailed written descriptions by several clinicians. The sample size was large enough to detect both overall and subgroup effects. Detailed data on folic acid use, as well as clinical records of pregnancy outcomes and external birth defects, allowed us to examine the associations among patterns of folic acid consumption, perinatal mortality compositions, and specific external major birth defects.

The main limitation of the data obtained from this public health campaign is the lack of randomization of folic acid supplementation. All women are advised to take 400 μg folic acid every day, and whether to follow the advice is up to their own ideas. However, we found additional adjustment for other demographic factors did not change the present results appreciably, meaning that the lack of randomization of folic acid supplementation induces few systematic differences between women who took folic use and those who did not. Second, maternal behaviors, such as smoking, drinking alcohol, or taking other nutritional supplements, have been confirmed to be potential confounders for perinatal mortality (38, 39). However, data on these risk factors were not collected at the time of the campaign. Further studies are needed to collect the relevant information to analyze their potential impact. Third, almost all participants in our study were of Han (China’s predominant ethnic group) ethnicity, as well as different diet patterns, living environment and lifestyles in the north and south may have been at play, which should be given a high-priority in future research.

## 5 Conclusion

We conclude that daily maternal consumption of 400 μg of folic acid without other vitamins had reduced peri- and neonatal mortality in China, especially in the northern region, and has potential to decrease major external birth defects and neural tube defect. Our findings support the importance of folic acid supplementary during pregnancy to enhance effective intervention of perinatal mortality. The results should be interpreted cautiously due to the limitations in this study. In the future, there is an urgent need for deep understandings of the role of folic acid in internal birth defect, the potential effect of other confounders, and the pathophysiology of adverse outcomes; all these will facilitate to reveal the mechanisms of fetus death and birth defect associated with folic acid.

## Data availability statement

The raw data supporting the conclusions of this article will be made available by the authors, without undue reservation.

## Ethics statement

The studies involving humans were approved by the Ethics Committee of the United States Centers for Disease Control and Prevention and the Peking University Health Science Center (grant no. U01DD000293). The studies were conducted in accordance with the local legislation and institutional requirements. The participants provided their written informed consent to participate in this study.

## Author contributions

XJL: Conceptualization, Writing – original draft. XWL: Data curation, Software, Writing – review & editing. HA: Data curation, Writing – review & editing. ZL: Supervision, Writing – review & editing. LZ: Visualization, Writing – review & editing. YZ: Validation, Writing – review & editing. JL: Software, Writing – review & editing. RY: Supervision, Writing – review & editing. NL: Conceptualization, Writing – original draft, Writing – review & editing.
