# Supplementary material for: Folic acid supplements and perinatal mortality in China
Source: Front Nutr. 2024 Jan 8;10:1281971. doi: 10.3389/fnut.2023.1281971 (PMC10800445; doi:10.3389/fnut.2023.1281971)
Supplement: Supplementary file 1 [file Table_1.DOCX]

**Supplementary Table 1. The number and frequency of major external birth defects among 222,303 singleton pregnant women in China, 1993 to 1996.**

| **Major external birth defects** | **Number** | **Frequency (‰)** |
| --- | --- | --- |
| **Anencephaly** | **65** | **0.29** |
| **Craniorachischisis** | **43** | **0.19** |
| **Iniencephaly** | **2** | **0.01** |
| **Spina Bifida** | **94** | **0.42** |
| **Encephalomyelocele** | **3** | **0.01** |
| **Encephalocele** | **32** | **0.14** |
| **Microcephaly** | **22** | **0.10** |
| **Congenital Hydrocephalus** | **173** | **0.78** |
| **Holoprosencephaly Face** | **14** | **0.06** |
| **Absence/Hypoplasia of Eye** | **13** | **0.06** |
| **Congenital Cataract** | **8** | **0.04** |
| **Absence/Hypoplasia of Ear** | **90** | **0.40** |
| **Accessory Auricle** | **810** | **3.64** |
| **Cleft Palate Only** | **77** | **0.35** |
| **Cleft Lip Only** | **132** | **0.59** |
| **Cleft Lip+Palate** | **223** | **1.00** |
| **Cleft Lip+/-Palate** | **355** | **1.60** |
| **Imperforate Anus** | **48** | **0.22** |
| **Persistent Cloaca** | **3** | **0.01** |
| **All External Genitalia Defects** | **122** | **0.55** |
| **All Polydactyly** | **300** | **1.35** |
| **Syndactyly** | **56** | **0.25** |
| **Reduction Defect, Any Limb** | **85** | **0.38** |

**Supplementary Table 2. Timing of and compliance with folic acid supplementation and risk of perinatal mortality without external major birth defects in China,** **1993 to 1996 per 1000 births.**

| Folic acid use | All cases of perinatal mortality without external major birth defects in the north | | | | |  | All cases of perinatal mortality without external major birth defects in the south | | | | |
| --- | --- | --- | --- | --- | --- | --- | --- | --- | --- | --- | --- |
|  | No. | Rate | Crude RR  (95% CI) | Adjusted RR  (95% CI)^a^ | |  | No. | Rate | Crude RR  (95% CI) | | Adjusted RR  (95% CI)^a^ |
| Timing^b^ |  |  |  | |  |  |  |  |  |  | |
| Periconception | 11,916 | 13.7 | 0.64 (0.53, 0.79) | | 0.69 (0.56, 0.86) |  | 54,012 | 13.5 | 0.82 (0.74, 0.91) | 0.87 (0.79, 0.97) | |
| Preconception | 3371 | 23.4 | 1.11 (0.86, 1.44) | | 1.18 (0.91, 1.54) |  | 32,495 | 14.0 | 0.86 (0.77, 0.97) | 0.91 (0.81, 1.03) | |
| Postconception | 1556 | 16.7 | 0.79 (0.53, 1.19) | | 0.81 (0.54, 1.23) |  | 15,022 | 14.4 | 0.89 (0.76, 1.05) | 0.94 (0.80, 1.11) | |
| Compliance |  |  |  | |  |  |  |  |  |  | |
| < 70% | 1643 | 14.0 | 0.66 (0.43, 1.01) | | 0.69 (0.45, 1.07) |  | 4325 | 12.9 | 1.00 (0.77, 1.31) | 1.06 (0.81, 1.39) | |
| 70% to < 90% | 3819 | 17.0 | 0.80 (0.61, 1.06) | | 0.85 (0.64, 1.13) |  | 12,843 | 10.7 | 0.83 (0.70, 0.99) | 0.88 (0.74, 1.05) | |
| ≥ 90% | 11,409 | 15.8 | 0.74 (0.61, 0.90) | | 0.81 (0.65, 0.99) |  | 84,426 | 10.9 | 0.85 (0.77, 0.92) | 0.90 (0.82, 0.98) | |
| No., number of pregnancies; RR, risk ratio; CI, confidence interval. | | | | | | | | | | | |
| ^a^Adjusted for maternal age (continuous), BMI (continuous), education, occupation, ethnicity and parity.  ^b^The analysis excluded 28 and 66 women in the northern and southern regions, respectively, for whom the timing of folic acid use could not be classified.  **Supplementary Table 3. Association of folic acid use with perinatal mortality composition without external major birth defects in China, 1993 to 1996 per 1000 births.**   \|  \| North \| \| \| \| \| \| \|  \| South \| \| \| \| \| \| \| --- \| --- \| --- \| --- \| --- \| --- \| --- \| --- \| --- \| --- \| --- \| --- \| --- \| --- \| --- \| \| Folic acid use \| Stillbirth \| \| \| Early neonatal death \| \| Neonatal death \| \|  \| Stillbirth \| \| Early neonatal death \| \| Neonatal death \| \| \| No. \| \| Rate \| No. \| Rate \| No. \| Rate \|  \| No. \| Rate \| No. \| Rate \| No. \| Rate \| \| None \| 11,668 \| \| 13.2 \| 11,514 \| 8.0 \| 11,514 \| 9.9 \|  \| 90,405 \| 7.3 \| 89,748 \| 5.7 \| 89,748 \| 7.4 \| \| Use \| 16,871 \| \| 11.6 \| 16,675 \| 4.3 \| 16,675 \| 5.8 \|  \| 101,594 \| 6.4 \| 100,944 \| 4.6 \| 100,944 \| 6.3 \| \| RR  95% CI \| \| 0.88  0.71, 1.09 \| \| 0.54  0.40, 0.73 \| \| 0.58  0.44, 0.76 \| \|  \| 0.88  0.79, 0.98 \| \| 0.81  0.72, 0.92 \| \| 0.85  0.76, 0.95 \| \| \| Adjusted RR  95% CI^a^ \| \| 0.95  0.75, 1.20 \| \| 0.57  0.41, 0.80 \| \| 0.64  0.48, 0.87 \| \|  \| 0.95  0.85, 1.06 \| \| 0.85  0.74, 0.96 \| \| 0.89  0.80, 0.99 \| \| \| No., number of pregnancies; RR, risk ratio; CI, confidence interval.  ^a^Adjusted for maternal age (continuous), BMI (continuous), education, occupation, folic acid use, ethnicity, and parity. \| \| \| \| \| \| \| \| \| \| \| \| \| \| \| | | | | | | | | | | | |

**Supplementary Table 4. Timing of and compliance with folic acid supplementation and risk of perinatal mortality without neural tube defects in China, 1993 to 1996 per 1000 births.**

| Folic acid use | All cases of perinatal mortality without neural tube defects in the north | | | | |  | All cases of perinatal mortality without neural tube defects in the south | | | | |
| --- | --- | --- | --- | --- | --- | --- | --- | --- | --- | --- | --- |
|  | No. | Rate^a^ | Crude RR  (95% CI) | Adjusted RR  (95% CI)^a^ | |  | No. | Rate^a^ | Crude RR  (95% CI) | | Adjusted RR  (95% CI)^a^ |
| Timing^b^ |  |  |  | |  |  |  |  |  |  | |
| Periconception | 11,972 | 15.3 | 0.62 (0.51, 0.75) | | 0.69 (0.56, 0.84) |  | 54,383 | 13.1 | 0.84 (0.77, 0.92) | 0.91 (0.82, 0.99) | |
| Preconception | 3390 | 24.5 | 1.00 (0.78, 1.28) | | 1.10 (0.85, 1.42) |  | 32,703 | 13.4 | 0.86 (0.77, 0.96) | 0.92 (0.82, 1.02) | |
| Postconception | 1562 | 19.2 | 0.78 (0.53, 1.14) | | 0.84 (0.57, 1.23) |  | 15,119 | 13.8 | 0.89 (0.77, 1.03) | 0.95 (0.82, 1.10) | |
| Compliance |  |  |  | |  |  |  |  |  |  | |
| < 70% | 1655 | 15.7 | 0.64 (0.43, 0.95) | | 0.69 (0.46, 1.04) |  | 4345 | 14.7 | 0.95 (0.74, 1.23) | 1.01 (0.79, 1.30) | |
| 70% to < 90% | 3836 | 18.5 | 0.75 (0.58, 0.98) | | 0.82 (0.62, 1.08) |  | 12,939 | 13.8 | 0.89 (0.76, 1.05) | 0.96 (0.82, 1.12) | |
| ≥ 90% | 11,461 | 17.4 | 0.71 (0.59, 0.85) | | 0.79 (0.65, 0.96) |  | 84,987 | 13.2 | 0.85 (0.79, 0.92) | 0.91 (0.84, 0.99) | |
| No., number of pregnancies; RR, risk ratio; CI, confidence interval. | | | | | | | | | | | |
| ^a^Adjusted for maternal age (continuous), BMI (continuous), education, occupation, ethnicity, and parity.  ^b^The analysis excluded 28 and 66 women in the northern and southern regions, respectively, for whom the timing of folic acid use could not be classified. | | | | | | | | | | | |

**Supplementary Table 5. Association of folic acid use with perinatal mortality composition without neural tube defects in China, 1993 to 1996 per 1000 births.**

|  | North | | | | | | |  | South | | | | | |
| --- | --- | --- | --- | --- | --- | --- | --- | --- | --- | --- | --- | --- | --- | --- |
| Folic acid use | Stillbirth | | | Early neonatal death | | Neonatal death | |  | Stillbirth | | Early neonatal death | | Neonatal death | |
|  | No. | | Rate | No. | Rate | No. | Rate |  | No. | Rate | No. | Rate | No. | Rate |
| None | 11,779 | | 15.3 | 11,599 | 9.3 | 11,599 | 11.8 |  | 91,063 | 8.6 | 90,283 | 7.0 | 90,283 | 8.8 |
| Use | 16,952 | | 12.3 | 16,744 | 5.3 | 16,744 | 6.7 |  | 102,271 | 7.7 | 101,480 | 5.6 | 101,480 | 7.6 |
| RR  95% CI | | 0.80  0.66, 0.98 | | 0.56  0.42, 0.75 | | 0.56  0.44, 0.72 | |  | 0.90  0.82, 0.99 | | 0.81  0.72, 0.91 | | 0.86  0.78, 0.95 | |
| Adjusted RR  95% CI | | 0.90  0.72, 1.13 | | 0.60  0.44, 0.82 | | 0.64  0.48, 0.84 | |  | 0.97  0.88, 1.08 | | 0.86  0.76, 0.96 | | 0.91  0.82, 1.01 | |
| No., number of pregnancies; RR, risk ratio; CI, confidence interval.  ^a^Adjusted for maternal age (continuous), BMI (continuous), education, occupation, folic acid use, ethnicity, and parity. | | | | | | | | | | | | | | |

Supplementary Table 6. Association of folic acid use with major external birth defects and central nervous system defects in China, 1993 to 1996 per 1000 births.

| Folic acid use | North (N=28,829) | |  | South (N=193,474) | |
| --- | --- | --- | --- | --- | --- |
|  | Major external birth defects  No. Rate | All central nervous system defects  No. Rate |  | Major external birth defects  No. Rate | All central nervous system defects  No. Rate |
| None | 187 15.77 | 90 7.59 |  | 36 8.08 | 145 1.59 |
| Use | 103 6.07 | 33 1.94 |  | 739 7.22 | 136 1.33 |
| Crude RR | 0.38 | 0.26 |  | 0.89 | 0.84 |
| 95% CI | 0.30, 0.49 | 0.17, 0.38 |  | 0.81, 0.99 | 0.66, 1.06 |
| Adjusted RR ^a^ | 0.52 | 0.43 |  | 0.93 | 0.94 |
| 95% CI | 0.40, 0.69 | 0.27, 0.67 |  | 0.84, 1.03 | 0.74, 1.20 |
| ^a^ Adjusted for maternal age (continuous), BMI (continuous), education, occupation, ethnicity, and parity. | | | | | |
